# Associations between survey completion mode and sociodemographic factors among individuals eligible for lung cancer screening

**DOI:** 10.1017/cts.2025.10193

**Published:** 2025-12-03

**Authors:** Catherine S. Nagawa, Reid Anctil, Jordan Neil, Iván Flores, Natalie Durieux, Ruosi Shao, Yuchiao Chang, Elyse Park, Gina R. Kruse

**Affiliations:** 1 TSET Health Promotion Research Center, Stephenson Cancer Center, The University of Oklahoma Health Sciences Center, Oklahoma City, OK, USA; 2 Hudson College of Public Health, The University of Oklahoma Health Science Center, Oklahoma City, OK, USA; 3 Massachusetts General Hospital, Boston, MA, USA; 4 School of Communication, Florida State University, Tallahassee, FL, USA; 5 Harvard Medical School, Boston, MA, USA; 6 Division of General Internal Medicine, University of Colorado School of Medicine, Aurora, CO, USA

**Keywords:** Lung cancer screening, survey mode preference, mail-based surveys, health disparities, recruitment methods

## Abstract

**Background:**

To enhance representation in LCS research, we examined associations between participant characteristics and their preferred mode of survey completion among individuals eligible for LCS.

**Methods:**

Between February 2023 and April 2024, prospective participants were identified using electronic healthcare records from Mass General Brigham and the University of Oklahoma Health Sciences (OUHSC). We offered three modes of survey completion: online, by mail, or by phone. Eligible participants were 50–80 years old, had smoked within the past 15 years, and spoke English or Spanish. We used multinomial logistic regression to estimate relative risk ratios (RRR).

**Results:**

Outreach to 2,822 individuals resulted in a sample of 315 participants. The mean age was 61.7 years (SD = 10.9). Most respondents were women (63.0%) and identified as White (63.3%), 29.8 % were Hispanic. The most common survey completion mode was mail (37.1%), followed by online (35.9%) and phone (27.0%). Characteristics associated with completion by mail were study site (RRR = 6.86, 95%CI:3.10–15.14), and race (RRR = 3.63, 95%CI:1.53–8.61); with respondents at OUHSC or who did not identify as White being more likely to choose mail over online modality. Characteristics associated with phone completion, included older age (RRR = 1.11, 95% CI: 1.03–1.20), Spanish language preference (RRR = 9.28, 95%CI:2.38–36.09), and with local government or community insurance (RRR = 9.91, 95% CI:1.92–51.3).

**Conclusion:**

The current trend toward online surveys may not fully account for individual preferences for LCS research engagement, and could limit the representativeness in LCS studies if offline alternatives are not offered.

## Introduction

Lung cancer remains the leading cause of cancer-related mortality in the United States, accounting for nearly one in five cancer deaths nationwide [1,2]. Screening for lung cancer using low-dose computed tomography improves the likelihood of early detection, significantly reducing late-stage diagnoses where treatment options are often limited or less effective [3]. However, screening rates remained low at approximately16% in 2022 among the 13.5 million individuals eligible for screening under the updated US Preventive Services Task Force guidelines [4]. Screening rates varied across states (8.6%–28.7%), with disparities by race, ethnicity, and socioeconomic status (SES) [4].

Subgroups experiencing disparities in lung cancer screening (LCS) are often underrepresented in LCS implementation research [5]. For instance, the National Lung Cancer Screening Trial had a low representation of minority populations, with 90% of participants identified as White [6]. Smaller studies investigating individuals’ attitudes and priorities in real-world LCS practices show similar trends in sociodemographic sample distributions [7,8]. Underrepresentation in LCS research limits our ability to build an evidence base that can inform equitable LCS implementation processes. Improving representativeness in LCS research is essential for uncovering and addressing disparities along the LCS pipeline, including barriers to adherence, perceptions regarding LCS, and post-LCS follow-up care [9]. Greater attention should be given to designing recruitment protocols that maximize participation among subgroups underrepresented in LCS research.

Offering multiple modes of survey completion (e.g., by mail, online, by phone) improves response rates [10–12]. Major national surveys, such as American Community Survey [13], offer multiple modes of completion, accommodating preferences that have been shown to vary by sociodemographic factors such as gender and age. Compared to women, men prefer responding to surveys by mail rather than in-person [14], and young adults prefer online methods, whereas older adults tend to prefer offline options [15,16]. The use of multiple survey modes in LCS research holds potential for improving representation of subgroups experiencing LCS disparities. However, preferences for survey completion based on language, location, and smoking status remain underexplored in the literature, despite their strong connection to key factors driving LCS disparities, such as race, state of residence, and SES. Addressing this gap could provide critical insights for tailoring recruitment methods to better engage underrepresented populations.

In this paper, we examined the associations between survey completion preferences and participant characteristics such as language, location, smoking status, and sociodemographic characteristics. Data were obtained from a two-site observational study that investigated factors influencing LCS intentions (main study results reported elsewhere). Participants were recruited from Massachusetts and Oklahoma, completing the survey either online, by mail, or by phone.

## Methods

### Study aims, setting, and population

This study was conducted between February 2023 and April 2024. We identified potential participants using electronic healthcare records (EHR) from two primary care systems: (1) Mass General Brigham (MGB) integrated healthcare delivery system that includes Massachusetts General Hospital and Brigham and Women’s Hospital primary care populations, and (2) The University of Oklahoma (OUHSC) primary care network. Massachusetts General Hospital consists of 18 primary care practices with 200 primary care physicians who care for ∼200,000 patients, and Brigham and Women’s Hospital consists of 15 primary care practices with nearly 200 primary care physicians who serve ∼163,000 patients. The Oklahoma Physicians Research Network currently has over 300 clinicians, from 137 primary care practices, serving nearly 10% of Oklahoma’s population (∼400,000).

We conducted a preliminary eligibility review for individuals who had indicated interest in participating in research on their EHR. Preliminary eligibility included: (1) age between 50 to 80 years, (2) a language preference for English or Spanish as indicated on their EHR, (3) not deceased (vital status), and (5) history of smoking. Eligibility was further assessed among individuals at the point of outreach to confirm smoking pack-years (≥15 years) using self-reported measures. In this initial assessment, we excluded individuals who were: (1) up to date with LCS based on their record in the past two years, (2) scheduled for a lung CT for diagnostic or abnormal follow-up evaluation, or (3) unable to consent to the study due to psychiatric or cognitive impairment as indicated on their record. Based the on preliminary EHR review, we identified 6,424 potentially eligible individuals across the two study sites. Our target sample size was 300. All study methods received approval from the institutional review boards of both MGB (IRB # 2021P003139), and OUHSC (IRB #14825) prior to the identification, initial contact, and recruitment of participants.

### Initial outreach

Initial outreach procedures varied slightly between the MGB and OUHSC systems due to institutional practices and differences in electronic patient communication systems. At the MGB site, participants were initially contacted through the patient portal or by mail, with portal users contacted electronically and non-users contacted by mail. Patient portals are secure online tools that allow patients to manage appointments, view health records, and explore clinical trial opportunities [17]. At the OUHSC site, all initial outreach was made via mail.

Beginning in February 2023, we implemented a batch outreach strategy to engage potentially eligible participants. Each week, we sent study materials via mail or email to ∼177 randomly selected individuals at MGB. At OUHSC, individuals were randomly selected within strata based on rurality, as defined by Rural–Urban Commuting Area (RUCA) codes [18]. The batch outreach strategy allowed us to efficiently manage outreach efforts and ensure adequate time for participant follow-up. Study materials were provided in both English and Spanish, and included information on the study aims, eligibility criteria, remuneration, risks/benefits to participation, and instructions for completing the survey or opting out of the study. Participants received either paper versions of the consent and survey form or a link to their electronic equivalents. We also provided study phone numbers and emails and encouraged individuals to contact us with any questions about the study.

### Follow-up procedures

**Figure 1.**
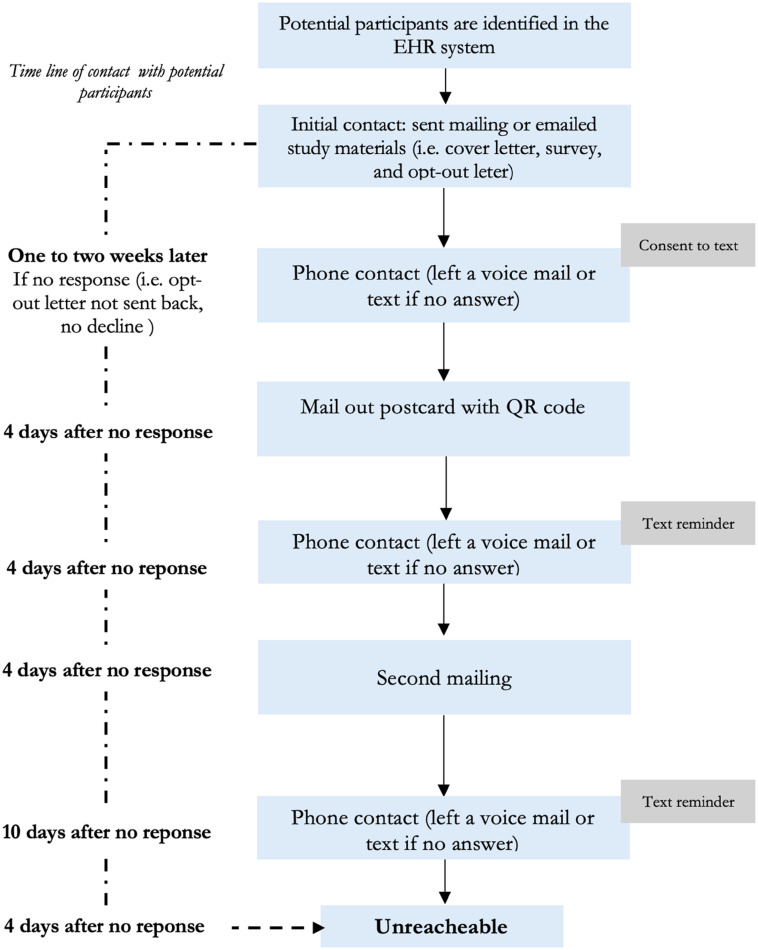
Follow-up procedures across for a lung cancer screening survey across two sites in Massachusetts and Oklahoma.

We used similar procedures in subsequent follow-up contacts across the two study sites. Two weeks following the initial contact (made by mail or electronically via the patient portal), individuals who had not opted out of the study were contacted by phone. During the first phone call, we provided information about the study and an option to complete the survey by phone or online. At MGB, participants reached by phone were also asked to confirm or deny consent to receive study text reminders. At the OUHSC site, obtaining consent to receive study text reminders was not required based on institutional protocols. Four days after the initial phone call, we mailed a postcard to all potential participants. The postcard included a QR code linking to the survey. Up to three additional follow-up attempts were made within the following 22 days, which included up to two phone calls (with text messages sent for OUHSC recruits and MGB recruits who consented to receive) and one additional mailing of the survey. Subsequent outreach was only conducted if the survey had not been received by the study team, and the participant had not contacted study staff to opt out of the study. Up to five contacts could be made before a participant was deemed unreachable. The total number of attempts made before survey completion was recorded for each participant. We achieved our target sample size in April 2024. (Figure 1).

### Data collection

Participants were offered three options to complete the survey: online, by mail, or by phone. The survey was available in both English and Spanish across all modes to ensure accessibility [19]. The estimated completion time was approximately 20–30 minutes. The reading level of the survey was assessed and written at a 6th-grade level to ensure accessibility. Participants responded to 50–70 questions, based on skip patterns. Data were collected in REDCap, and respondents received a $25 gift card. In the survey, we included questions to confirm study eligibility. Participants were asked: “Are you between the ages of 50 and 80 years old?,” “Have you smoked at least 100 cigarettes (5 packs = 100 cigarettes)?,” and “Have you smoked cigarettes in the last 15 years?” We included individuals who confirmed: (1) an age between 50 and 80 years, and (2) a history of smoking with pack-years ≥ 15. Individuals who did not meet these criteria were excluded from the study.

Eligible participants provided information on specific age, gender, race, ethnicity, preferred language (English or Spanish), marital status, education level, employment status, health insurance coverage, smoking status, and overall health. Smoking status was self-reported and recorded as formerly smoked or currently smoking.

### Statistical analysis plan

We calculated the recruitment rate as the percentage of the total number of participants recruited over the total number of individuals contacted. Using survey data, we provided descriptive statistics of individuals who participated and their proportions by survey completion modes (online, mail, or by phone). We used bivariate analysis to assess sociodemographic differences by survey completion mode. Chi-square tests were used to evaluate differences in categorical variables, and a one-way analysis of variance test for continuous variables. Multinomial logistic regression was used to examine multivariable associations between survey completion modes and sociodemographic factors. Variables were included in the multivariable model if there was established relevance in the literature (gender, age, and smoking status) or if they had a p-value less than 0.05 in the bivariate analysis. Multicollinearity was assessed using variance inflation factors (VIF) [20]. In our study, ethnicity and language preference were identified as highly collinear, and therefore ethnicity was excluded to improve model precision. Estimates of relative risk ratios (RRR) and 95% confidence intervals (CI) are presented. This analysis was conducted in STATA V18.

## Results

### Recruitment and response rate

**Figure 2.**
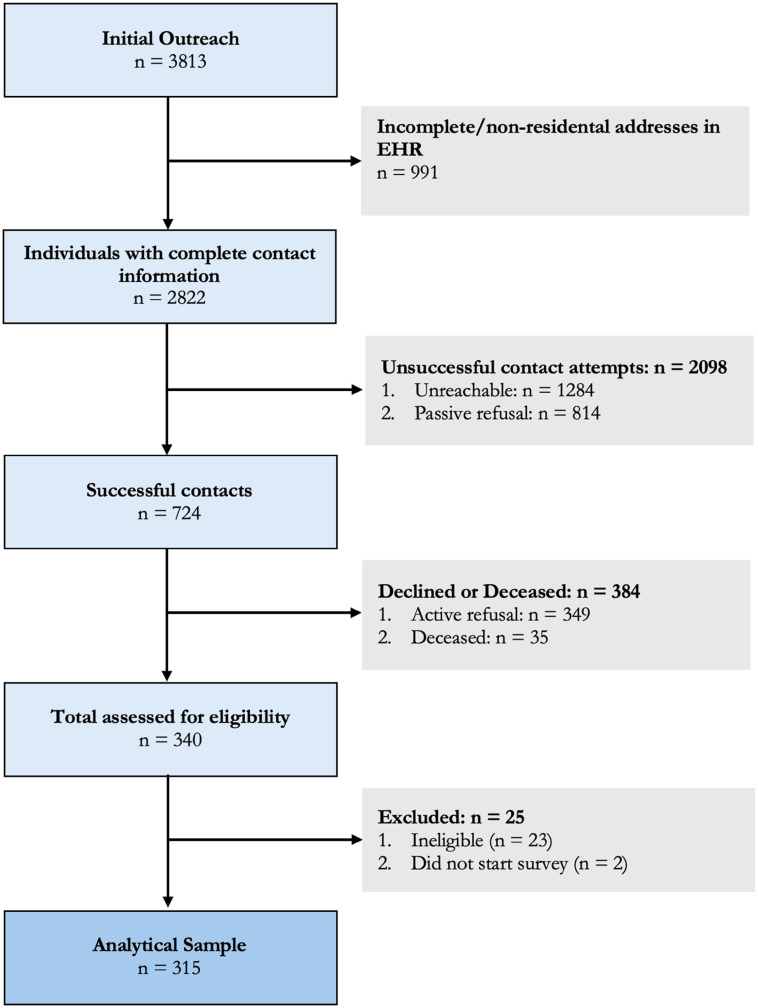
Flow diagram for participant recruitment in a lung cancer screening survey using electronic health records across two sites in Massachusetts and Oklahoma.

Out of the original EHR-identified sample pool, we excluded 991 individuals due to incomplete addresses or addresses associated with nursing homes, detention centers, or homeless shelters. Outreach attempts were made to the remaining 2,822 individuals prior to achieving the desired sample size. Among the 2,822 individuals, 45.5% (*n* = 1,284) were excluded due to incorrect contact information or invalid phone numbers in the EHR, 28.9% (*n* = 814) did not respond to any of our outreach attempts (passive refusals), and 25.7% (*n* = 724) were successfully contacted. Of the 724 contacted, 48.3% (*n* = 349) declined to participate (active refusals), 4.8% (*n* = 35) were reported deceased, and 47.0% (*n* = 340) were considered as eligible. Among the 340 eligible individuals, 6.8% (*n* = 23) were deemed ineligible after further screening, and 0.6% (*n* = 2) did not start the survey. The final sample size consisted of 315 participants. (Figure 2)

Of the 2,822 individuals with complete contact information, we excluded 1,342 from the recruitment denominator due to the following reasons: 35 were deceased, 23 were ineligible, and 1,284 were unreachable. This left 1,480 individuals eligible for recruitment.

The overall recruitment rate was 21.3% (315 out of 1,480). Of the 315 respondents, 191 were enrolled at MGB and 124 at the University of Oklahoma Health Sciences Center (OUHSC). The site-specific recruitment rates were 15.2% for MGB (191 out of 1,254) and 55.1% for OUHSC (124 out of 225).

### Participant characteristics and survey completion modes

The mean age of participants was 61.7 years (SD = 10.9). Most respondents were women (*n* = 199, 63.0%), identified as White (*n* = 190, 63.3%), and were non-Hispanic (*n* = 221, 70.2%). Table 1 displays the characteristics of the study population.

**Table 1.** Characteristics of lung cancer screening eligible individuals who participated in the lung cancer screening study, recruited in Oklahoma and Massachusetts (*n* = 315)

Participant characteristics (*n* = 315)		*N* (%)
Study site	MGB	191 (60.6)
	OUHSC	124 (39.4)
Age, *mean (SD)*		61.7 (6.9)
Gender	Man	115 (36.6)
	Woman	199 (63.4)
Race	White	190 (63.3)
	Multiple races	39 (13.0)
	Black or African American	36 (12.0)
	Other*	35 (11.7)
Ethnicity	Hispanic	94 (29.8)
	Non-Hispanic	221 (70.2)
Preferred language	English	226 (71.7)
	Spanish	89 (28.3)
Marital status	Married	123 (39.2)
	Divorced	91 (29.0)
	Single or widowed	100 (31.8)
Education	Less than high school graduate or GED	155 (49.7)
	Post high school training or associate degree	91 (29.2)
	Bachelor’s degree, graduate, professional	66 (21.2)
Employment	Employed (full and part-time)	99 (31.4)
	Unable to work due to disability	127 (40.3)
	Retired	69 (21.9)
	Provide homecare, are unemployed, unknown	20 (6.3)
Health insurance	Employer/individual private/military	74 (23.6)
	insurance	68 (21.7)
	Medicare	101 (32.3)
	Medicaid or other public state program	58 (18.5)
	Local government or community insurance uninsured/don’t know/other	12 (3.8)
Smoking status	Formerly smoked	131 (41.7)
	Currently smokes	183 (58.3)
Overall health	Poor or fair	159 (50.5)
	Good, very good, excellent	156 (49.5)

* Other race includes Asian (*n* = 1), American Indian or Alaska Native (*n* = 6), Unknown (*n* = 28); GED: General Educational Development.

Missing: Race (*n* = 15); Education (*n* = 3); Marital status (*n* = 1), Education (*n* = 3); age (*n* = 1); Smoking status (*n* = 1).

**Figure 3.**
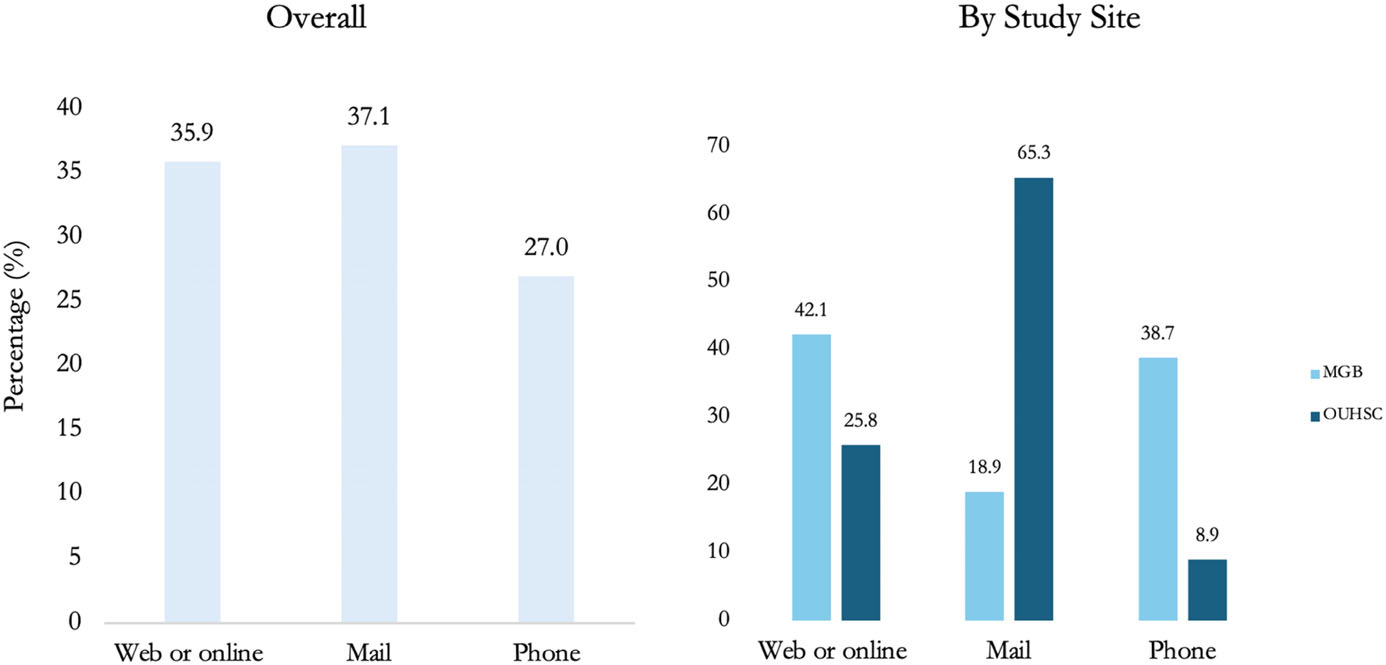
Overall and site-specific percent distribution of survey completion modes among eligible individuals recruited in Oklahoma and Massachusetts (*n* = 315).

The most common mode of survey completion was mail (37.1%), followed by the online (35.9%) option. Participants who enrolled at MGB were more likely to complete the survey online (42.4%) or by phone (38.7%), with mail being the least preferred option (18.9%). In contrast, participants at OUHSC showed a strong preference for mail (65.3%), with the phone as the least likely option (8.9%). The differences in survey response modes between the two sites were significant (*p* < 0.001) (Figure 3).

### Bivariate analysis of sociodemographic factors by survey completion mode

Table 2 shows differences in sociodemographic factors by survey completion mode. The mean age of participants was lowest in those who completed the survey online (mean age: 59.8 years, SD = 6.5) and highest in those who completed by phone (mean age: 63.1 years, SD = 7.7) (*p* < 0.001). Men were more likely to complete the survey by mail, while women were more likely to use the online mode (*p* < 0.001).

**Table 2.** Participant sociodemographic factors by survey completion modes among lung cancer screening eligible individuals recruited in Oklahoma and Massachusetts (*n* = 315)

Participant characteristics^#^		Online *n* = 113 (35.9%)	Mail *n* = 117 (37.1%)	Phone *n* = 85(27.0%)	*P*-value
Study site	MGB	81 (42.4)	36 (18.9)	74 (38.7)	<0.001
	OUHSC	32 (25.8)	81 (65.3)	11 (8.9)	
Age, *mean (SD)*		59.8 (6.5)	62.7 (6.4)	63.1 (7.7)	<0.001
Gender	Man	39 (33.9)	51 (44.4)	25 (21.7)	0.11
	Woman	73 (36.7)	66 (33.2)	60 (30.2)	
Race	White	94 (49.5)	74 (38.95)	23 (11.6)	<0.001
	Multiple races	6 (16.7)	14 (38.9)	16 (44.4)	
	Black or African American	4 (10.3)	15 (38.5)	19 (51.3)	
	Other*	6 (17.4)	6 (17.1)	23 (65.7)	
Ethnicity	Non-Hispanic	98 (44.3)	102 (46.2)	21 (24.7)	<0.001
	Hispanic	15 (16.0)	15 (16.0)	64 (68.0)	
Preferred language	English	100 (44.3)	105 (46.5)	21 (9.3)	<0.001
	Spanish	13 (14.6)	12 (13.5)	64 (71.9)	
Marital status	Married	45 (36.6)	52 (42.3)	26 (21.1)	0.011
	Divorced	35 (38.5)	37 (40.7)	19 (20.9)	
	Single or widowed	32 (32.0)	28 (28.0)	40 (40.0)	
Education	Less than high school graduate or GED	40 (25.8)	52 (33.6)	63 (40.7)	<0.001
	Post high school training or associate degree	34 (37.4)	43 (47.3)	14 (15.4)	
	Bachelor’s degree, graduate, professional	38 (57.6)	21 (31.8)	7 (10.6)	
Employment	Employed (full and part-time)	48 (48.5)	31 (31.3)	20 (20.2)	0.025
	Unable to work due to disability	35 (27.6)	49 (38.6)	43 (33.9)	
	Retired	23 (33.3)	31 (44.9)	15 (21.7)	
	Provide homecare, are unemployed, unknown	7 (35.0)	6 (30.0)	7 (35.0)	
Health insurance	Employer/individual private/military	40 (36.0)	28 (23.9)	6 (7.1)	<0.001
	Medicare	24 (21.6)	36 (30.8)	8 (9.4)	
	Medicaid or other public state program	41 (36.9)	39 (33.3)	21 (24.7)	
	Local government or community insurance	4 (3.6)	9 (7.7)	45 (52.9)	
	Uninsured/don’t know/other	2 (1.8)	5 (4.3)	5 (5.9)	
Smoking status	Formerly smoked	50 (38.2)	41 (31.3)	40 (30.5)	0.2
	Currently smokes	63 (34.4)	75 (41.0)	45 (24.6)	
Overall health	Poor or fair	45 (28.3)	57 (35.9)	57 (35.9)	<0.001
	Good, very good, excellent	68 (43.6)	60 (38.5)	28 (17.9)	
Number of contacts to participants, mean (SD)		2.8 (1.8)	2.6 (1.9)	3.2 (1.4)	0.03

* Other race includes Asian (*n* = 1), American Indian or Alaska Native (*n* = 6), Unknown (*n* = 28); GED: General Educational Development.

Missing: Race (*n* = 15); Education (*n* = 3); Marital status (*n* = 1), Education (*n* = 3); Age (*n* = 1); Smoking status (*n* = 1); ^#^row percentages are provided for categorical demographic characteristics.

Non-Hispanic respondents preferred to complete the survey either online (44.3%) or by mail (46.2%), with the least likely option being the phone (24.7%). In contrast, Hispanic respondents showed a higher preference for completing by phone (68.0%) (*p* < 0.001). Similar to ethnicity, language preferences showed comparable trends (*p* < 0.001). Other demographic factors that differed significantly by survey completion mode included marital status, education, and employment.

Completion mode did not differ by smoking status (*p* = 0.2). Participants who completed the survey by phone had the highest average number of outreach contacts (mean: 3.2, SD = 1.4), compared to online (mean: 2.8, SD = 1.8) and mail (mean: 2.6, SD = 1.9) (*p* = 0.03).

### Multivariable association between survey completion mode and sociodemographic factors

#### Mail vs. online

In the adjusted analysis, using online completion as the reference, we found that study site and race were associated with completion by mail rather than online. Respondents at the OUHSC site were six times more likely to opt for mail completion compared to those enrolled at MGB (RRR = 6.86, 95% CI: 3.10–15.14). Respondents who did not identify as White were significantly more likely to complete the survey by mail (RRR = 3.63, 95% CI: 1.53–8.61) compared to those who identified as White (Table 3).

**Table 3.** Relative risk estimates and 95% confidence interval for factors influencing survey completion modes among eligible individuals recruited in Oklahoma and Massachusetts (*n* = 315)

	Preferred mail over online mode RRR (95% CI)	Preferred phone over online mode RRR (95% CI)	Preferred phone over mail mode RRR (95% CI)
Study site			
MGB	1	1	1
OUHSC	6.86 (3.10–15.14)*	2.46 (0.76–8.01)	0.36 (0.11–1.12)
Age, mean (SD)	1.07 (1.00–1.13)	1.11 (1.03–1.20)*	1.04 (0.97–1.12)
Gender			
Man	1	1	1
Woman	0.67 (0.34–1.35)	1.14 (0.45–2.89)	1.69 (0.69–4.11)
Race**			
White	1	1	1
Other race*	3.63 (1.53–8.61)*	3.21 (1.07–9.71)*	0.88 (0.31–2.54)
Preferred language			
English	1	1	1
Spanish	0.72 (0.2–2.46)	6.64 (1.82–24.20)*	9.28 (2.38–36.09)*
Marital status			
Married	1	1	1
Divorced	0.66 (0.29–1.50)	0.36 (0.11–1.21)	0.54 (0.17–1.74)
Single or widowed	0.75 (0.33–1.72)	1.56 (0.55–4.40)	2.08 (0.76–5.67)
Education			
Less than high school graduate or GED	1	1	1
Post high school training or associate degree	1.17 (0.54–2.52)	1.74 (0.60–5.02)	1.49 (0.54–4.08)
Bachelor’s degree, graduate, professional	0.44 (0.19–1.06)	0.35 (0.09–1.46)	0.80 (0.19–3.39)
Employment			
Employed (full and part-time)	1	1	1
Unable to work due to disability	1.82 (0.70–4.74)	1.46 (0.43–5.01)	0.80 (0.24–2.92)
Retired	0.89 (0.30–2.64)	0.87 (0.19–3.98)	0.99 (0.23–4.29)
Provide homecare, are unemployed, unknown	1.00 (0.23–4.30)	1.64 (0.25–10.74)	1.64 (0.27–10.1)
Health insurance			
Employer/individual private/military	1	1	1
Medicare	0.78 (0.27–2.20)	0.94 (0.18–4.77)	1.22 (0.25–5.98)
Medicaid or other public state program	0.52 (0.18–1.48)	1.16 (0.26–5.24)	2.23 (0.49–10.1)
Local government or community insurance	2.68 (0.57–12.52)	26.5 (4.26–165.5)*	9.91 (1.92–51.3)*
Uninsured/don’t know/other	2.49 (0.36–17.46)	4.92 (0.52–46.1)	1.97 (0.28–13.63)
Smoking status			
Formerly smoked	1	1	1
Currently smokes	1.17 (0.61–2.28)	0.69 (0.28–1.68)	0.59 (0.25–1.39)
Overall health			
Good, very good, or excellent	1	1	1
Poor or fair	0.97 (0.48–1.96)	0.59(0.28–1.68)	0.61 (0.25–1.52)
Number of contacts to participants, mean (SD)	1.02 (0.84–1.25)	1.22 (0.95–1.68)	1.19 (0.93–1.52)

* p-value<0.01; **Race used as a binary variable: White (*n* = 190) and Other race (*n* = 110, (which in includes Multiple race (*n* = 39), Black or African American (*n* = 36), and those who checked off Other race in survey (*n* = 35)).

#### Phone vs. online

Those who did not identify as White also preferred using the phone more than the online option (RRR = 3.21, 95% CI: 1.07–9.71). We found that older participants, those who preferred the Spanish language, and those with local government or community insurance preferred completing the survey by phone rather than online. For each additional year in age, the likelihood of selecting the phone option increased by 11% (RRR = 1.11, 95% CI: 1.03–1.20). Compared to respondents with employer or individual private insurance, those insured through the local government or community programs were more likely complete the survey by phone (RRR = 26.5; 95% CI: 4.26–165.5) rather than online. Those who preferred Spanish were six times more likely to complete the survey by phone (RRR = 6.64, 95% CI: 1.82–24.20) (Table 3).

#### Phone vs. mail

Respondents who preferred Spanish also preferred completing the survey by phone over mail (RRR = 9.28, 95% CI: 2.38–36.09). Individuals with local government or community insurance preferred completing the survey by phone (RRR = 9.91, 95% CI: 1.92–51.3) compared to mail. Individuals recruited at OUHSC, those who preferred Spanish, and those with local government or community insurance favored completing the survey by mail over online and phone over mail (Table 3).

Gender, overall health, marital status, education, employment, smoking status, and the number of contacts showed no significant differences across survey completion modes.

## Discussion

We explored how survey completion preferences varied by sociodemographic characteristics to better understand response patterns across online, mail, and phone modes. Mail was the most preferred completion mode. Preferences for mail over the online mode was observed in individuals recruited from the Oklahoma site and those who did not identify as White. Non-white respondents also showed a stronger preference for phone over the online mode. Additional factors associated with phone survey completion included older age, having health insurance coverage through local government or community programs, and a preference for the Spanish language.

Even with a moderate recruitment rate of 21% [21], we achieved strong representation across key demographic groups, including Hispanic individuals (29%), Black or African American individuals (12%), individuals whose highest level of education less than a high school diploma or GED (49%), and those unable to work (40%). Nearly half of the respondents were individuals who currently smoke. In comparison, a similar study that conducted a survey to identify factors associated with LCS, interviewing patients by phone, recruited a sample that included 4% Black or African American individuals, 6% Hispanic individuals, and 25% with a high school education [22]. Our study also achieved greater representation of these subgroups compared to the National Lung Screening Trial (NLST) [23], which recruited participants from 33 medical institutions across the United States, with trained interviewers assessing eligibility through phone or in-person interviews. Although direct comparisons are not appropriate due to differences in methodologies and recruitment strategies, these findings highlight the effectiveness of our approach in engaging a diverse participant pool.

### Mail preference

Consistent with prior findings [24], we found that respondents who did not identify as White were more likely to prefer mail over online completion, compared to White respondents. Older individuals similarly favored mail, aligning with previous research demonstrating that older individuals are more likely to prefer non-digital options [15,16]. Online survey modalities are increasingly used in research due to their widespread accessibility, cost-effectiveness, and faster processing times [25–27]. However, offering offline survey completion modes in LCS research remains essential, particularly because (1) LCS eligibility criteria [28] skews towards older individuals, and (2) non-White populations are consistently underrepresented in LCS research [6–8]. Offline modes could bridge gaps in representation but may require careful consideration of resources to be factored into recruitment timelines and budgets, such as staff time for administering phone surveys and managing data entry for mail responses.

Respondents who were recruited at the Oklahoma site showed a strong preference for completing the survey by mail compared to those recruited at the Massachusetts site. While these differences were initially assumed to reflect regional or site-specific preferences, they seem to be more likely tied to the initial method of participant contact. At the Oklahoma site, all participants were initially contacted by mail, which likely contributed to the higher mail completion rates. In contrast, at the Massachusetts site, where initial outreach primarily occurred through the patient portal (i.e., email), majority of participants completed the survey online. This view suggests that initial outreach methods may influence how respondents engage and respond to surveys. The commonly used “push-to-web” approach, where all respondents are initially mailed an invitation to complete a survey online, followed by a paper survey for nonrespondents in a later mailing [29], may not fully support the recruitment of representative samples in LCS research. Offering all survey completion modes upfront, including online and paper formats, could encourage engagement in underrepresented groups as it allows respondents to select their preferred method from the outset.

### Phone preference

Respondents who preferred Spanish demonstrated a strong preference for completing surveys via phone. This mode may be particularly favored in this subgroup as it addresses difficulties in survey question comprehension commonly observed among Spanish-speaking respondents. Cho et al. found that Latino respondents born in Mexico or Puerto Rico who preferred communicating in Spanish experienced greater difficulty understanding survey questions [30]. These challenges, also noted by other researchers [31,32], are uniquely tied to cultural factors and likely contribute to the observed preference for phone surveys. The phone option enables participants to request immediate clarifications and improves their comprehension of survey questions. Offering a phone survey option may help address challenges in recruiting Spanish-speaking and Hispanic individuals, who often face disparities in accessing LCS and related healthcare services [9,33,34].

Participants insured through local or community programs may prefer phone surveys due to practical factors and/or unique experiences with healthcare systems. Programs such as Commonwealth Care Alliance in Massachusetts and Oklahoma Cares in Oklahoma provide targeted support to individuals with complex health needs or specific health conditions. Unlike private insurance (which is tied to employment), Medicaid (which covers low-income individuals), or Medicare (which predominantly serves older adults), this group encompasses a more diverse and dynamic population with distinct health and service-related needs. In our sample, individuals covered by these specialized insurance programs were older, often unable to work, reported poor overall health, and had lower educational attainment. Past studies show that vulnerable populations, including those with significant health and socioeconomic challenges, prefer offline modes [35].

### Limitations

This study has several limitations. Recruiting participants from the healthcare system likely resulted in a sample skewed toward individuals who were actively engaged in their healthcare, potentially limiting generalizability to those who face healthcare access barriers. Known challenges of using EHR data include outdated or incomplete contact information, missing health data, and an overrepresentation of insured individuals. Additionally, despite our efforts to recruit rural participants, our sample included few individuals (*n* = 8) from rural areas, and all respondents who indicated a preference for Spanish were enrolled at the MGB site. This may reflect limitations in our recruitment methods, which may not have effectively engaged these subpopulations.

## Conclusion

We observed a strong preference for offline survey completion modes (i.e., phone and mail) among subgroups that tend to be underrepresented in LCS research. The current trend toward online surveys may overlook individual preferences for LCS research engagement, potentially limiting study representativeness if offline alternatives are not offered. Providing multiple modes of survey completion revealed preferences for certain modes, and highlighted the relative importance of these preferences based on individuals’ characteristics. While participation in LCS research does not directly translate to screening uptake, our findings offer valuable insights [5] for engaging subgroups affected by LCS disparities to improve representativeness, ultimately strengthening the LCS evidence base to improve LCS uptake and better outcomes.

## Data Availability

The data underlying this study cannot be shared publicly or upon request due to ethical and privacy concerns. Specifically, the small sample sizes of protected groups included in the study raise the risk of participant re-identification. Summary data are provided within the manuscript, but individual-level data cannot be shared in order to protect participant confidentiality.
